# Robotic-Assisted Computed Tomography-Guided Percutaneous Bone Procedures: A Prospective Multicenter Pilot Study

**DOI:** 10.1007/s00270-026-04545-w

**Published:** 2026-07-29

**Authors:** Baptiste Bonnet, Nicolas Stacoffe, Michael Dassa, Lambros Tselikas, Paul Beunon, Sean Tutton, Jean-Baptiste Pialat, Laurent Milot, Louis Thiery, Charles Mastier, Nassima Daidj, William Boulade, Thierry de Baère, Frédéric Deschamps, Gilles Piana

**Affiliations:** 1 Department of Anesthesiology, Surgery and Interventional Radiology, Gustave Roussy, 114 rue Édouard Vaillant, 94805 Villejuif, France; 2 Department of Radiology, Groupement Hospitalier Sud, Hospices Civils de Lyon, Pierre-Bénite, France; 3 Interventional Radiology Department, Institut Paoli Calmettes, Aix Marseille University, 232 Boulevard Sainte Marguerite, 13009 Marseille, France; 4 Department of Radiology, UC San Diego, San Diego, CA USA; 5 Department of Diagnostic and Interventional Radiology, Hôpital Edouard Herriot, Hospices Civils de Lyon, Lyon, France

**Keywords:** Robotic assistance, Computed tomography, Spine, Bone intervention, Cementoplasty, Percutaneous procedure

## Abstract

**Purpose:**

To prospectively evaluate the feasibility, safety, and targeting accuracy of robotic-assisted CT-guided percutaneous bone procedures.

**Materials and Methods:**

This prospective multicenter pilot study included adult patients undergoing CT-guided percutaneous bone consolidation or tumor ablation between September 2024 and January 2025 at three academic institutions. All procedures were planned and performed using the Epione® robotic platform. Targets included the thoracic and lumbar spine and the pelvis. The primary endpoint was feasibility of robotic-assisted needle insertion, defined as successful needle placement without conversion to manual technique. Secondary endpoints included safety, targeting accuracy, procedural time, and radiation dose. Targeting accuracy was measured as lateral tip deviation and angular deviation between planned and final needle positions.

**Results:**

Thirty-four patients underwent 90 needle insertions, including 66 in the spine (73.3%) and 24 in the pelvis (26.7%). Indications were bone consolidation in 87/90 insertions (97%) and tumor ablation in 3/90 (3%). Robotic insertion was feasible in 89/90 insertions (98.9%). Seven adverse events occurred in 6 patients (17.6%), including four serious events, none related to the device or procedure. Mean lateral tip deviation was 2.0 ± 1.4 mm, and mean angular deviation was 3.1° ± 2.7°. Mean dose–length product was 3224 ± 2215 mGy.cm. Mean total procedure time was 03 h 09 min ± 50 min.

**Conclusions:**

Robotic-assisted CT-guided bone procedures showed high feasibility and satisfactory targeting accuracy, with no device- or procedure-related adverse events. These findings support further evaluation in larger comparative studies.

**Clinical Trial Registration:**

NCT06376682.

**Graphical Abstract:**

**Figure:**
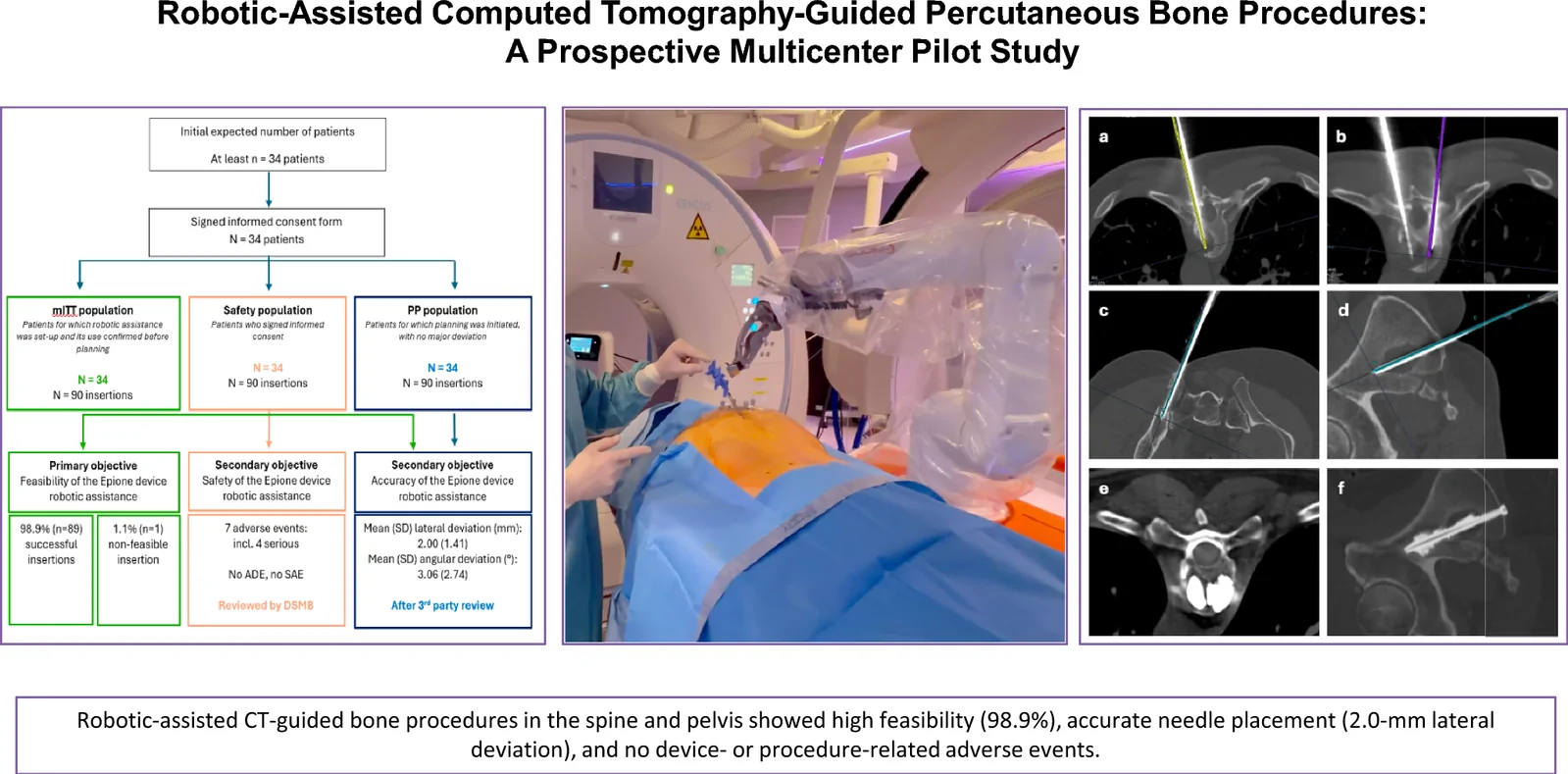

**Supplementary Information:**

The online version contains supplementary material available at https://doi.org/10.1007/s00270-026-04545-w.

## Introduction

Interventional radiology has undergone major advances over the past decade, largely driven by improvements in image guidance and navigation technologies [1, 2]. Robotic platforms have emerged as promising tools to enhance the precision, reproducibility, and safety of percutaneous procedures [3]. Their use has already been evaluated in abdominal and thoracic applications, with promising results for needle placement accuracy, particularly in complex or high-risk anatomical settings [4, 5].

Percutaneous bone interventions are increasingly used in oncologic and non-oncologic settings. In oncology, image-guided procedures such as ablation, vertebral augmentation, cementoplasty, and percutaneous fixation play an important role in the management of painful or unstable bone metastases, with prevalence rates reaching up to 80% in patients with advanced cancers [6, 7]. As systemic therapies prolong survival, the demand for repeatable, precise local treatments continues to grow [8]. Beyond oncology, minimally invasive consolidation procedures are also increasingly performed for osteoporotic and traumatic fractures, reflecting the growing need for low morbidity treatments in fragile patients [9, 10]. The spine represents a major target for these procedures, with the pelvis sharing similar technical challenges in terms of access planning and proximity to critical structures [11, 12].

Despite their clinical value, percutaneous spinal and pelvic procedures remain technically demanding. Needle insertion may be particularly difficult for double-oblique trajectories, narrow osseous corridors, or approaches performed close to neural and vascular structures [12, 13]. In addition, repeated CT guidance may increase radiation exposure for both patients and operators [14].

To address these limitations, several navigation systems, including electromagnetic and cone-beam CT-based guidance systems, have been evaluated in spinal and pelvic interventions. Although these technologies have yielded promising results, they remain limited by external tracking, the absence of respiratory synchronization, and continued dependence on manual needle advancement [15–18]. Robotic guidance may overcome some of these limitations by enabling reproducible trajectory planning and controlled needle insertion [19]. While preliminary clinical data have been reported for robotic-assisted CT-guided bone biopsies, evidence remains scarce for robotic assistance in complex bone consolidation and ablation procedures [20].

A robotic platform previously validated for abdominal and thoracic applications has recently been adapted for musculoskeletal interventions [21]. Following preclinical validation, the present multicenter pilot study was designed to prospectively evaluate the feasibility, safety, and targeting accuracy of robotic-assisted CT-guided percutaneous bone procedures, with a particular focus on spinal and pelvic interventions.

## Materials and Methods

### Study Design, Outcomes, and Endpoints

**Fig. 1:**
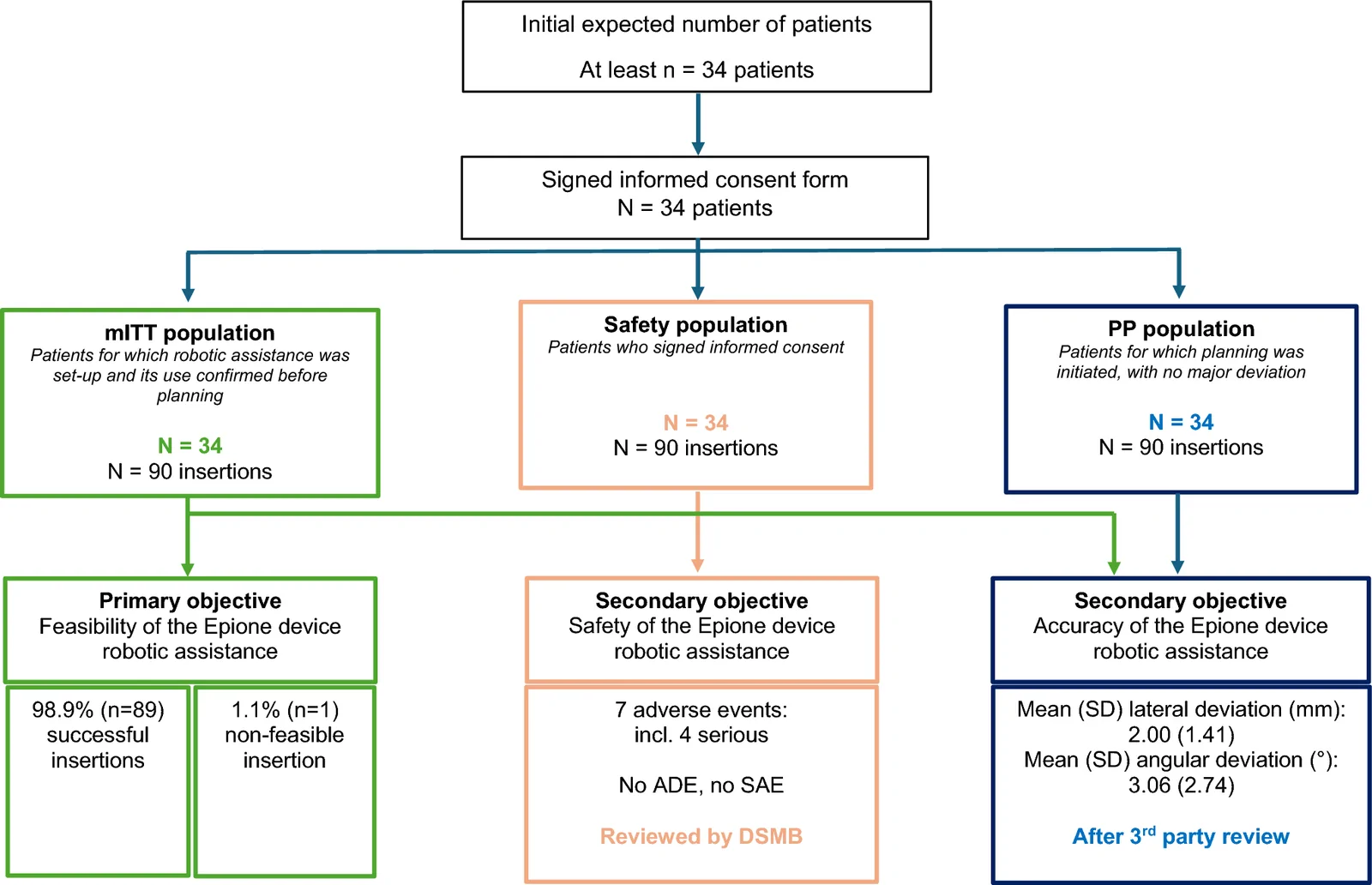
Study flowchart. Flow diagram of patient enrollment, analysis populations, and study endpoints, including the modified intention-to-treat, per-protocol, and safety populations

This prospective, multicenter, single-arm pilot study was conducted at three academic centers in France between September 2024 and January 2025. The primary objective was to assess the feasibility of robotic-assisted needle insertion during CT-guided percutaneous bone procedures. The primary endpoint was technical success, defined as the successful completion of needle placement without conversion to conventional manual technique. Conversion was recorded in cases of device malfunction, insufficient targeting accuracy as assessed by the operator on control CT, or any procedural circumstance requiring manual completion in the interest of patient safety (Fig. 1).

Secondary outcomes included safety, targeting accuracy, procedure time and radiation dose. All adverse events (AEs) occurring during the 1-month follow-up period were recorded, classified according to the modified Cardiovascular and Interventional Radiological Society of Europe (CIRSE) classification system, and adjudicated for their potential relationship to the device or procedure [22]. Targeting accuracy was assessed by measuring both lateral tip (mm) and angular (°) deviations between the planned trajectory and the final needle position on post-placement CT. Accuracy measurements were independently reviewed and, when necessary, corrected after secondary image registration review.

### Patients

Eligible participants were consecutive adult patients scheduled for a CT-guided percutaneous bone procedure under general anesthesia. Indications were validated according to local multidisciplinary decision-making procedures, including multidisciplinary tumor board review for oncologic cases when applicable. Exclusion criteria were procedures involving the skull or cervical spine, contraindications to general anesthesia, inadequate respiratory control, pregnancy, and breastfeeding.

Written informed consent was obtained from all participants before enrollment. The study was conducted in accordance with Good Clinical Practice and applicable regulatory requirements. It was approved by the French national ethics committee (Comité de Protection des Personnes Est 1, approval number SI 24.00781.000307) and by the coordinating center’s Institutional Review Board (approval number IRB n°3946).

### Procedure

All procedures were performed under general anesthesia using the Epione® robotic platform (Quantum Surgical, Montpellier, France). The study included the initial cohort of patients undergoing bone procedures performed with the robotic device, after dedicated operator training, corresponding to the early phase of the learning curve. As previously published, the robotic workflow was adapted for bone procedures by stiffening the needle guide and implementing a dedicated two-step workflow to ensure stable cortical anchoring before final advancement [21]. An intermediate CT acquisition was systematically performed after cortical penetration, allowing robotic recapture and trajectory adjustment when needed (Supplementary Data 1).

As procedures frequently required the insertion of multiple needles through a narrow entry zone, the robotic system incorporated a collision avoidance feature that detected previously placed needles and optimized both the robotic arm movements and needle insertion sequence.

All procedures were performed using diamond-tip trocars, ranging from 8 to 13 gauge, with harmonization of the material across centers before study initiation. Needle caliber was selected according to the procedure type, target location, and osseous dimensions; 8-gauge trocars were used for percutaneous osteosynthesis.

Procedures were performed by 14 board-certified interventional radiologists with 5–20 years of experience. Most, but not all, radiologists had previous experience with the studied robotic system in soft tissues. All investigators completed dedicated training on the robotic platform and its bone-specific workflow before participation in the study (Supplementary Data 2).

### Secondary Outcomes Assessment

Safety was monitored intraoperatively and during the 1 month postoperative follow-up. Final assessment was based on clinical follow-up and cross-sectional imaging performed 4–6 weeks after the intervention (CT, MRI, or other modalities as clinically indicated). AEs were recorded and graded according to the modified CIRSE classification system [22]. An independent Data and Safety Monitoring Board (DSMB) convened twice (once after completion of patient enrollment and again at the end of follow-up) to review all AEs and adjudicate their potential relationship to the device or procedure.

Targeting accuracy was assessed using robotic software-based registration between the planning CT and the post-placement control CT. Lateral tip deviation and angular deviation were calculated from the planned and actual coordinates of the needle tip and shaft. All measurements were independently reviewed by a senior interventional radiologist (S.T., > 20 years of experience). When registration correction was necessary, updated accuracy values were computed.

Challenging trajectories were predefined as insertions located in the upper thoracic spine (T1–T4) or requiring trans-ischiatic/posterior column or ilio-pubic access. These trajectories were considered challenging because of their marked z-axis obliquity, limited accessibility using a standard bull’s-eye approach, and narrow osseous corridors [23].

### Data Collection

Demographic, clinical, and procedural data were prospectively collected in standardized electronic case report forms (ShareCRF S.L., Madrid, Spain). Collected variables included age, sex, clinical indication, lesion location and type, number of needle insertions, intraoperative technical data, radiation dose, and procedural timings. Accuracy data (planned and actual needle trajectories) were extracted from CT images, DICOM files, and robotic software logs on the day of the procedure. Source data were anonymized and stored in a secure centralized database. Data monitoring and consistency checks were performed according to the study monitoring plan.

### Statistical Considerations

Statistical analyses were performed, according to a predefined statistical analysis plan by a biostatistician (ICUREsearch, France) using the SAS® software (v9.4). Additional subgroup analyses were performed afterward and considered exploratory.

Sample size was based on the primary endpoint of technical feasibility assessed at the needle insertion level. Using a one-sample non-inferiority design with Cohen’s arcsine transformation, and assuming a feasibility rate of 97% based on preclinical data with a non-inferiority margin of 0.08, at least 67 insertions were required to achieve 90% power with a one-sided alpha of 0.05 [24, 25]. Assuming a mean of approximately two insertions per patient in routine practice, 34 patients were planned for inclusion.

The primary endpoint was reported with a two-sided 95% confidence interval (CI). Quantitative variables are presented as means ± standard deviation or median (range), as appropriate, and qualitative variables as counts and frequencies (%). Missing data were not imputed.

To account for the non-independence of multiple trajectories performed in a single patient, subgroup comparisons of targeting accuracy were assessed using linear mixed-effects models with patient identifier as a random effect. Fixed effects included predefined clinical subgroups: (i) spine versus pelvis procedures, (ii) pedicular versus costotransverse approaches, (iii) challenging versus non-challenging trajectories, and (iv) metastatic versus osteoporotic fractures. Both lateral and angular deviations were analyzed separately. *P*-values < 0.05 were considered statistically significant.

## Results

### Patients

Thirty-four consecutive patients were enrolled between September and November 2024, with follow-up completed in January 2025, including 20 women (58.8%) and 14 men (41.2%), with a mean age of 64.3 (± 14.5) years. Eighteen patients (52.9%) were receiving concurrent oncologic treatment at the time of the procedure, most commonly chemotherapy (11/18, 61%). Among metastatic bone lesions, the most frequent primary tumors were breast cancer (15/45, 33.3%) and lung cancer (10/45, 22.2%). At baseline, 26/34 (76.5%) patients reported pain at the treatment site and were receiving analgesic treatment. Six patients (17.6%) received antiresorptive therapy (Table 1).

**Table 1:** Patients’ characteristics

Variables	Total (*n* = 34 patients)
Gender, n (%)	
Men	14/34 (41%)
Women	20/34 (59%)
Age (years), mean (± SD)	64.3 (± 14.5)
Body mass index, mean (± SD)	24.4 (± 4.4)
Concurrent oncologic treatment (%)	18/34 (53%)
Radiotherapy	5/18 (28%)
Chemotherapy	11/18 (61%)
Immunotherapy	3/18 (17%)
Analgesic therapy, n (%)	26/34 (77%)
Level 1	6/26 (23%)
Level 2	7/26 (27%)
Level 3	13/26 (50%)
Antiresorptive therapy (%)	6/34 (18%)

### Procedures

**Fig. 2:**
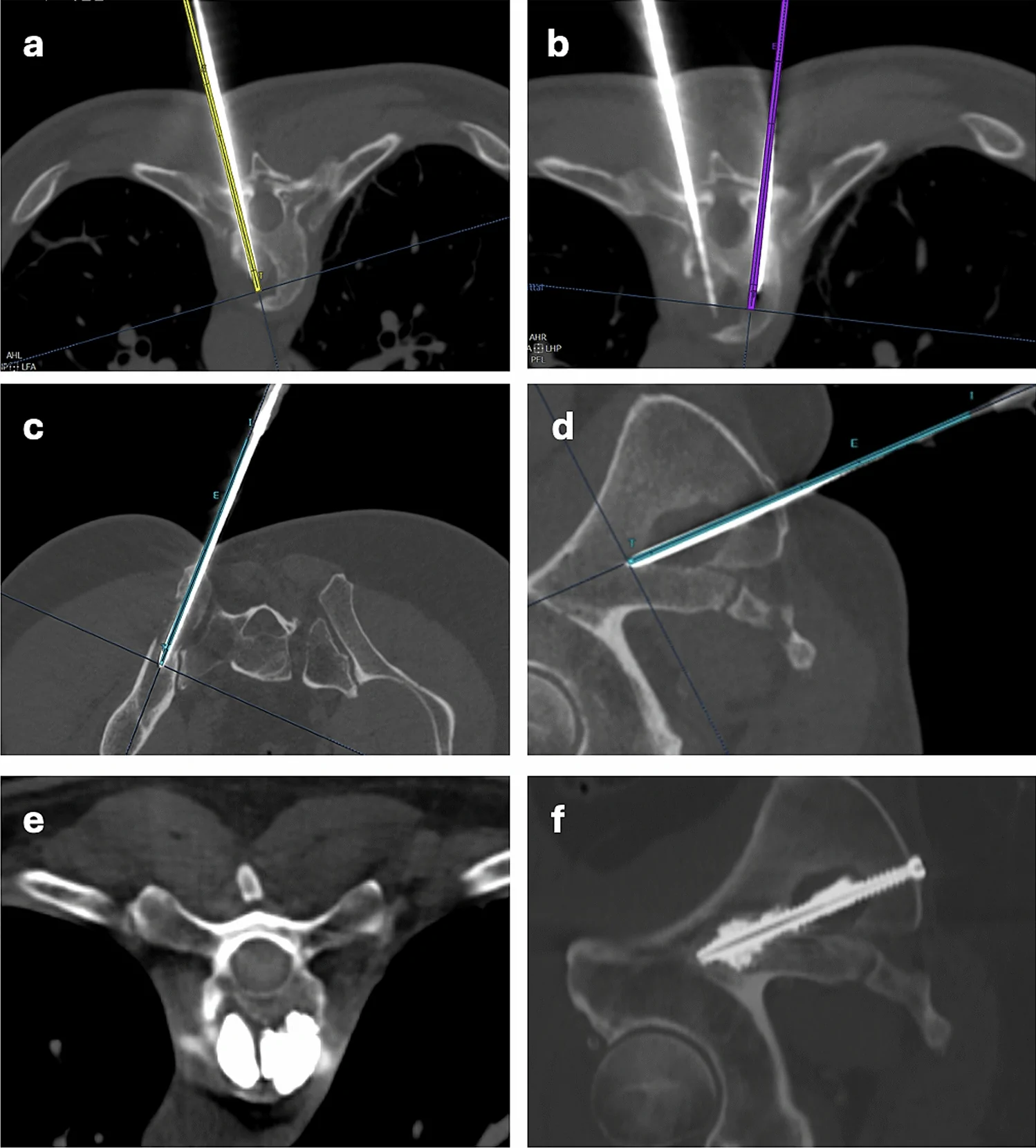
Representative case of combined spinal and pelvic consolidation. A 55-year-old patient with renal cell carcinoma receiving systemic therapy with cabozantinib presented with lytic bone metastases at risk of fracture involving the T8 vertebral body and the left iliac wing. The patient underwent T8 vertebroplasty and cement-augmented fixation of the left iliac wing. (**a**, **b**) Intraoperative axial CT images at T8 showing bilateral pedicular access with cementoplasty trocars aligned with the planned robotic trajectories (yellow and violet). (**c**, **d**) Intraoperative axial and oblique sagittal CT images of the pelvis showing trocar placement through the iliac lesion via a posterior approach, aligned with the planned trajectory (light blue). (**e**) Post-procedural axial CT image at T8 showing satisfactory cement distribution within the vertebral body. (**f**) Post-procedural oblique sagittal CT image of the pelvis showing satisfactory screw placement across the iliac lesion with distal cement anchoring

**Fig. 3:**
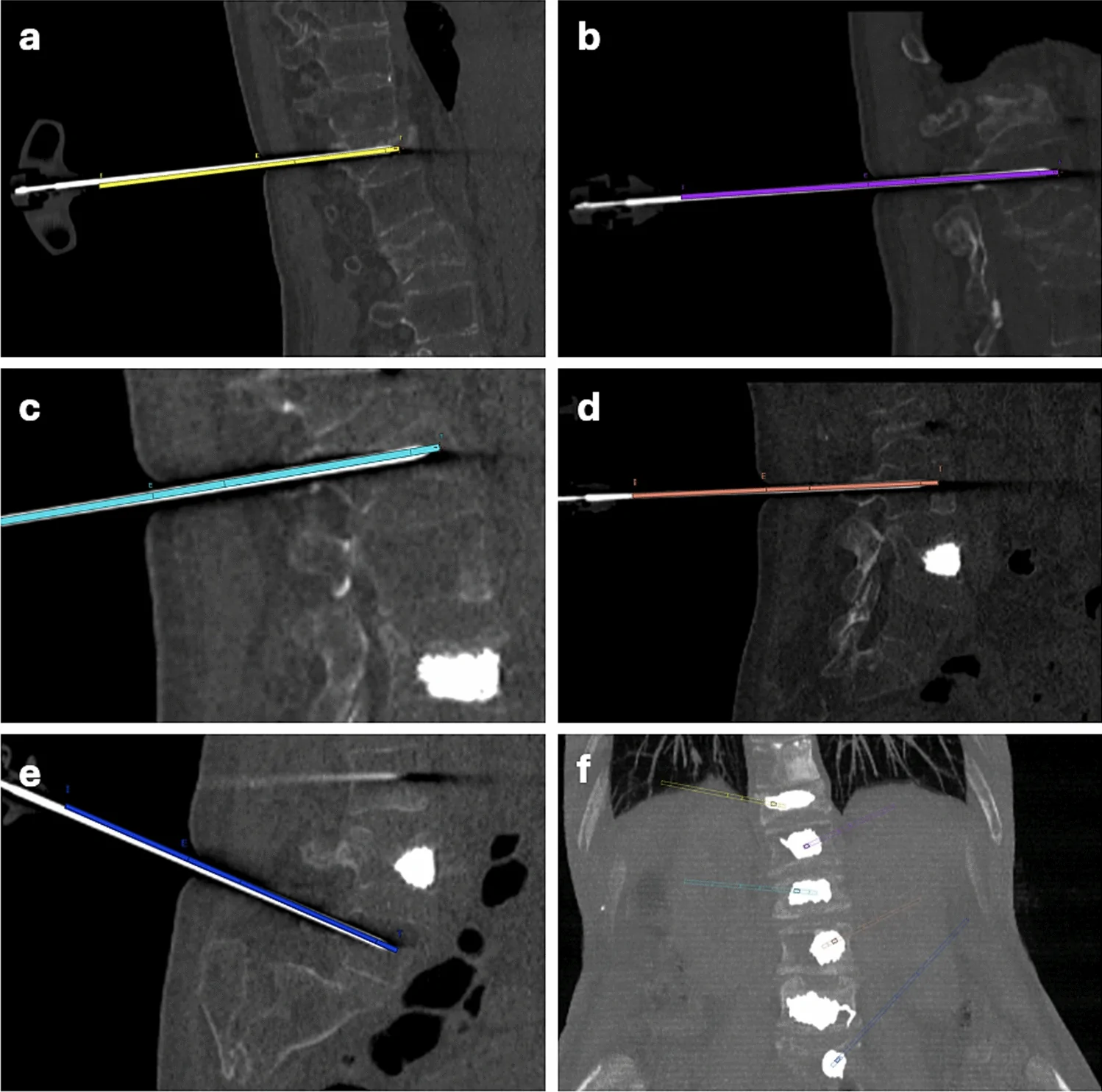
Representative case of multilevel vertebral consolidation. A 49-year-old woman with metastatic bone disease from a thymic neuroendocrine tumor presented with multiple thoracolumbar vertebral fractures involving T12 to L5, associated with mixed metastatic and osteoporotic involvement and severe disabling pain. The patient had previously undergone vertebroplasty at L4 and presented with new compression fractures at T12-L3 and L5. Multilevel vertebroplasty was performed for analgesic and consolidation purposes. (**a**-**e**) Intraoperative oblique sagittal CT images obtained in the plane of the pedicular trajectories at T12-L3 and L5, with superimposed planned robotic trajectories (colored lines). (**f**) Coronal maximum intensity projection reconstruction from the post-procedural CT showing satisfactory cement distribution across the treated vertebral bodies

A total of 90 needle insertions were performed, corresponding to a mean of 2.6 insertions per patient (range, 1–7). Targets were located in the spine for 66/90 needles (73.3%), mainly at the thoracic level (43/66, 65.2%), and in the pelvis for 24/90 needle insertions (26.7%), mostly in the sacrum (17/24, 71%). Among the 90 insertions, 87/90 (96.7%) were performed for bone consolidation procedures, including osteoplasty in 71/87 cases (81.6%) and osteosynthesis in 19/87 cases (21.8%) (three combined procedures). The remaining 3/90 insertions (3.3%) were performed for tumor ablation procedures, including two cryoablation and one electrochemotherapy procedure. The most frequent access routes were pedicular for spinal procedures (52/66, 78.8%) and trans-sacroiliac for pelvic procedures (17/24, 71%). Additional details regarding indications, lesion types, and access routes are provided in Table 2. Two patient cases are illustrated in Figs. 2 and 3.

**Table 2:** Procedural data

Variables	Total (*n* = 90 needles)
Number of needles per patient, mean (± SD)	2.6 (± 1.6)
Procedure type, *n* (%)	
Bone consolidation	87/90 (97%)
Osteoplasty	71/87 (82%)*
Osteosynthesis	19/87 (22%)*
Ablation	3/90 (3%)
Cryoablation	2/3 (67%)
Electrochemotherapy	1/3 (33%)
Associated biopsy	3/90 (3%)**
Location, *n* (%)	
Spine	66/90 (73%)
Upper thoracic	9/66 (14%)
Other thoracic	34/66 (52%)
Lumbar	23/66 (35%)
Pelvis	24/90 (27%)
Sacrum	17/24 (71%)
Iliac wing	7/24 (29%)
Lesion size (mm), mean (SD)	28.7 (± 21.8)
Indication, *n* (%)***	
Bone tumors	49/90 (54%)
Bone metastasis	45/49 (92%)
Primary tumor	4/49 (8%)
Radiological type of tumors	
Osteolytic	41/49 (84%)
Osteoplastic	1/49 (2%)
Mixed	7/49 (14%)
Fractured bone	54/90 (60%)
Oncologic fractures	26/54 (48%)
Osteoporotic fractures	25/54 (46%)
Traumatic fractures	3/54 (6%)
Needle size (gauge), *n* (%)	
13	32/90 (36%)
11	41/90 (46%)
8	17/90 (19%)
Guidewire used, *n* (%)	11/90 (12%)
Access route, *n* (%)	
Spine	
Pedicular track	52/66 (79%)
Costotransverse track	14/66 (21%)
Pelvis	
Trans-sacroiliac track	17/24 (71%)
Anterior trans-iliac track	4/24 (17%)
Posterior trans-iliac track	1/24 (4%)
Anterior ilio-pubic track	1/24 (4%)
Sacral track	1/24 (4%)
Procedure timing, mean (SD), hh:min	
Total duration (patient-in to patient-out)	03:09 (00:50)
Robotic system use (planning and needle insertion)	00:53 (00:34)
Dose–length product, mean (SD), mGy cm	3224 (± 2215)

^*^Three combined procedures

^**^The three biopsies were combined with two spine bone consolidation procedures and with one pelvic ablation

^***^Categories not mutually exclusive

The mean dose–length product was 3224 ± 2215 mGy cm. The mean total procedure time was 03 h 09 min ± 50 min, and the mean duration of needle planning and insertion was 53 ± 34 min.

### Feasibility

Robotic-assisted needle placement was successfully achieved in 89/90 insertions (98.9%; 95% CI, 94%–100%), corresponding to 33/34 treated patients (Table 3). The single technical failure occurred during treatment of an osteolytic pelvic metastasis from renal cell carcinoma, requiring conversion to manual placement after cortical slippage and persistent targeting inaccuracy despite two robotic attempts.

**Table 3:** Feasibility and accuracy outcomes of robotic-assisted procedures

Variables	Total (*n* = 34 patients and *n* = 90 needles)
Feasibility	
Success rate per patient	33/34 (97%)
Success rate per needle	89/90 (99%) [95% CI: 93.9–99.9%]
Accuracy*	
Accuracy, mean (± SD)	
Lateral deviation (mm)	2.0 (± 1.5)
Angular deviation (°)	3.1 (± 2.8)
Trajectories that required image registration revision after independent review, n (%)	7/89 (8%)
Accuracy post-review, mean (± SD)	
Lateral deviation (mm)	2.0 (± 1.4)
Angular deviation (°)	3.1 (± 2.7)
Insertion achieved without cortical needle reinsertion, n (%)	87/89 (98%)
Modifications of the needle trajectory after intermediate CT scan at cortical anchorage, *n* (%)	
None	60/89 (67%)
1	28/89 (32%)
More than 1	1/89 (1%)
Additional CT scan control, *n* (%)	
None	19/29 (66%)
1	9/29 (31%)
More than 1	1/29 (3%)

^*^Data are presented on the 89 feasible needles

### Safety

A total of seven adverse events occurred in 6/34 patients (17.6%) during the 1 month evaluation period. Four events met criteria for serious adverse events, including two deaths attributed to oncologic progression and two prolonged hospitalizations related to respiratory distress and chemotherapy-related heart failure. After independent DSMB review, none of the events were considered related to the robotic device or the procedure. Individual adverse events, modified CIRSE grades, adjudicated relationship, and outcomes are detailed in Table 4.

**Table 4:** Safety data: all adverse events according to the CIRSE classification

Adverse events	*n*	Time from procedure	Modified CIRSE grade	Serious AE	Relationship to device/procedure	Outcome
Pain without neurological deficit	1	Within follow-up	Grade 1a	No	Not related	Resolved
Altered general condition, nausea, or vomiting	1	Day of procedure	Grade 2	No	Not related	Resolved
Acute dyspnea in the context of decompensated heart failure	1	Within follow-up	Grade 2	No	Not related	Resolved
Respiratory distress requiring prolonged hospitalization	1	Within follow-up	Grade 3a	Yes	Not related	Resolved
Chemotherapy-related heart failure requiring prolonged hospitalization	1	Within follow-up	Grade 3a	Yes	Not related	Resolved
Clinical deterioration attributed to oncologic progression	2	Within follow-up	Grade 6	Yes	Not related	Death

^*^AE, Adverse events

^**^Adverse events were graded according to the modified CIRSE classification (Filippiadis et al. 2026)

### Accuracy

The mean lateral deviation between the planned and actual needle position was 2.00 mm (± 1.50 mm) at the needle tip, and the mean angular deviation was 3.1° (± 2.8°). Following independent third-party review, image registration was corrected for 7/89 trajectories (7.8%). After correction, updated mean lateral deviation was 2.00 mm (± 1.41 mm) and a mean angular deviation of 3.1° (± 2.7°).

Among the 89 successful robotic insertions, 2/89 (2.2%) required cortical reinsertion. In 60/89 cases (67.4%), the needle reached the target at first attempt, without any robotic-assisted trajectory modification after the mandatory intermediate CT. In 28/89 insertions (31.5%), a single robotic trajectory adjustment was required before successful target access. One additional insertion (1.1%) required six robotic trajectory adjustments due to iterative cortical slippage.

Among the 29 insertions requiring a trajectory adjustment, 19/29 (65.5%) were completed without any CT acquisition beyond the mandatory intermediate control. Nine insertions (31%) required one additional CT acquisition, and one insertion (3.4%) required six scans, corresponding to the same case that required six successive trajectory adjustments.

### Subgroup Analyses

Exploratory subgroup analyses showed no significant difference in targeting accuracy according to lesion location (spine vs pelvis), spinal access route (pedicular vs costotransverse), clinical indication (metastatic vs osteoporotic), or trajectory complexity (challenging vs non-challenging) (Supplementary Table 1).

## Discussion

This prospective multicenter pilot study showed that robotic-assisted CT-guided percutaneous bone procedures were feasible and safe, with high technical success and consistent targeting accuracy. Robotic-assisted needle placement was successful in 98.9% of insertions, with mean lateral and angular deviations of 2.0 mm and 3.1°, respectively. No adverse events were attributed to the robotic device or to the procedure after independent DSMB review.

The single technical failure occurred in an osteolytic pelvic metastasis involving the ilio-pubic branch, where the entry zone was constrained between two narrow cortices and cortical slippage prevented stable progression along the planned trajectory. Similar anchoring difficulties are encountered in manual pelvic procedures, particularly when narrow osseous corridors must be accessed precisely [26]. This case highlights the importance of selecting a stable cortical entry point during pre-procedural planning. Based on this limited experience, flatter and more regular cortical surfaces may be more favorable for robotic anchoring than crest-like or highly curved entry zones. However, because only one technical failure occurred, no formal framework for entry zone selection can be derived from the present study. Successful insertions were also achieved in other technically demanding situations, including costotransverse trajectories in the thoracic spine, suggesting that complex spinal access remains compatible with robotic guidance when the cortical entry conditions are adequate.

The feasibility rate observed in our study was consistent with previous reports of robotic assistance in thoracic and abdominal procedures [4, 5]. The two-step workflow used in this study was specifically developed for bone procedures, with initial cortical anchoring followed by intermediate CT control, robotic recapture, and trajectory adjustment when required. Although this workflow component was not evaluated as an independent endpoint, it was successfully integrated into the procedure workflow and did not compromise technical success. It may be particularly useful when cortical anchoring is uncertain or when minor angular correction is required before final advancement. Because two-thirds of successful insertions did not require trajectory modification after cortical anchoring, a simplified workflow may be worth evaluating in future clinical studies, especially for less complex trajectories.

All procedures were performed using diamond-tip trocars, selected to favor straight advancement through the robotic needle guide. In selected cases involving narrow or anatomically complex osseous corridors, alternative needle designs such as beveled-tip devices might facilitate cortical engagement or navigation between adjacent cortices. Future technical developments could, therefore, explore expanded needle compatibility to improve versatility in complex spinal and pelvic anatomy [27, 28].

Seven adverse events occurred in six patients during follow-up, including four serious adverse events; however, none was considered related to the robotic device or procedure after DSMB adjudication. This finding is reassuring given the clinical complexity of the cohort, which included frail patients undergoing oncologic or fracture-related interventions. Although the study was not designed to compare complication rates with manual techniques, the observed safety profile does not suggest any excess risk associated with robotic guidance.

Targeting accuracy was consistent across anatomical subgroups, with mean deviations within the range reported in prior studies of image-guided robotic interventions and spinal navigation systems [29–31]. The mean lateral deviation of approximately 2 mm should be interpreted in the context of the procedures performed. Most insertions were performed for bone consolidation, for which this degree of final tip deviation is unlikely to affect clinical safety or technical success when the trajectory remains within the planned osseous corridor. In the present cohort, no adverse event was related to targeting accuracy, and no significant difference in accuracy was observed between predefined challenging and non-challenging trajectories. Minor deviations were most likely related to cortical engagement on irregular bony surfaces or to mechanical stress during advancement through dense bone. The absence of a significant difference across spinal, pelvic, and predefined challenging trajectories suggests that the platform may provide reproducible targeting performance across a broad spectrum of technically demanding procedures.

Direct comparison with conventional freehand CT-guided procedures remains difficult, as freehand procedures are usually not based on a software-defined trajectory that can be systematically compared with the final needle position. Therefore, lateral and angular deviations between planned and actual trajectories are rarely available for freehand techniques. Direct comparison with robotic surgical platforms is also limited, because published studies often evaluate different endpoints, such as pedicle screw grading, three-dimensional tip deviation, or intraoperative revision rates, and are generally performed in open or hybrid surgical settings rather than in percutaneous CT-guided interventions. Nevertheless, the accuracy observed in the present study (approximately 2 mm lateral deviation and 3° angular deviation) appears broadly consistent with values reported in the spinal navigation literature [30, 31].

The present results should also be interpreted in light of prior studies on robotic or navigation-assisted musculoskeletal interventions. Witkowska et al. reported the feasibility and safety of a patient-mounted robotic system for CT-guided bone biopsies in oncologic patients [19]. However, their study focused on biopsies performed under local anesthesia, whereas the present study evaluated more complex procedures performed under general anesthesia, mainly multi-needle consolidation procedures in the spine and pelvis. Studies of cone-beam CT navigation for pelvic screw fixation and comparative CT- versus cone-beam CT-guided fixation provide relevant benchmarks, but differ substantially in imaging environment, procedure type, and workflow [17, 18]. These differences highlight the need for future comparative studies specifically designed to evaluate workflow efficiency and radiation exposure.

Reported procedure times and radiation doses vary substantially across studies, reflecting differences in indications, imaging guidance modalities, procedural complexity, and workflow definitions. In the present study, total procedure duration was comparable to, or slightly shorter than, previously reported durations for robotic-assisted microwave ablation, navigation-assisted fixation, and pelvic cementoplasty or screw fixation [16–18, 32]. In contrast, radiation exposure was higher than in several previous reports [16, 19, 32]. This likely reflects several factors specific to the present pilot workflow: First, a mandatory intermediate CT acquisition was performed after cortical anchoring for each needle as a safety check before final advancement; second, procedures frequently involved multiple needles, with a mean of nearly three insertions per patient; and third, many targets were located in the spine or pelvis, where z-axis coverage and automatic tube current modulation may increase dose–length product. These factors should be considered when interpreting dosimetric results, and future workflow optimization may reduce radiation exposure once procedural safety and feasibility have been established.

Future implementation in routine interventional radiology practice will require further workflow optimization. In particular, compatibility with cone-beam CT environments may be important, as cone-beam CT is widely used for spinal and musculoskeletal procedures. Radiation exposure may also decrease with experience and with refinement of the current two-step workflow, particularly if future studies identify situations in which intermediate CT controls can be safely reduced.

This study has several limitations. Its single-arm design, limited sample size, and lack of control group preclude any formal comparison with conventional CT-guided techniques. Cost data were not collected, and cost-effectiveness could not be assessed. In addition, the heterogeneity of indications and target locations may limit the generalizability of the findings and the interpretation of exploratory subgroup analyses. The study also focused on spinal and pelvic procedures. Extremity procedures were not included because percutaneous bone consolidation of long bones is less frequently performed in interventional radiology practice and remains more commonly managed surgically, particularly when load-bearing fixation is required.

Importantly, this series included the initial cases performed with the robotic system for bone interventions, corresponding to the early phase of the learning curve. Although all operators completed dedicated training before participation, this likely influenced several reported outcomes and may limit their representativeness compared with results achievable in routine practice once proficiency has been attained. Accordingly, procedural time and radiation exposure should be interpreted with caution, as both metrics may improve with operator experience and workflow optimization [33]. Finally, the current musculoskeletal workflow remains constrained by technical factors, including needle length and the position of the needle guide on the shaft, which may restrict access to some deep targets or reduce flexibility for complex trajectories. Further studies in larger and more homogeneous cohorts, together with ongoing technical refinements, are warranted.

## Conclusion

In this prospective multicenter pilot study, robotic-assisted CT-guided percutaneous bone procedures were highly feasible and demonstrated satisfactory targeting accuracy, with no device or procedure-related adverse events. These findings support further evaluation of this approach for spinal and pelvic bone interventions in larger comparative studies.

## Supplementary Information

Below is the link to the electronic supplementary material.

Supplementary file1 (TIFF 4304 KB)

Supplementary file2 (DOCX 14 KB)

Supplementary file3 (MP4 301116 KB)
