# Supplementary material for: Robotic-Assisted Computed Tomography-Guided Percutaneous Bone Procedures: A Prospective Multicenter Pilot Study
Source: Cardiovasc Intervent Radiol. 2026 Jul 29;49(9):1875–85. doi: 10.1007/s00270-026-04545-w (PMC13597657; doi:10.1007/s00270-026-04545-w)
Supplement: Supplementary file 1 [file 270_2026_4545_MOESM2_ESM.docx]

**Supplementary Table 1:** Accuracy subgroup analyses depending on the lesion characteristics

| **Subgroup** | **Lateral deviation**  (*p*-values) | **Angular deviation**  (*p*-values) |
| --- | --- | --- |
| Spine *vs* Pelvis | 0.66 | 0.78 |
| Pedicular *vs* Costo-transverse approach | 0.62 | 0.71 |
| Challenging *vs* Non-challenging | 0.14 | 0.20 |
| Oncological *vs* Osteoporotic fracture | 0.27 | 0.33 |
